# Proposing a revised Hanau's formula for determining lateral condylar guidance: A comparative study

**DOI:** 10.1016/j.jobcr.2025.08.014

**Published:** 2025-08-16

**Authors:** Jayant N. Palaskar, Amit D. Hindocha, Prashant D. Jadhav, Pooja P. Hasabnis

**Affiliations:** a Department of Prosthodontics, Sinhgad Dental College and Hospital, Pune, India

**Keywords:** Hanau's formula, Lateral condylar guidance, Cephalogram, lateral records, Articulator settings

## Abstract

**Background:**

Inaccurate lateral condylar guidance (LCG) recordings lead to occlusal interferences, increasing patient discomfort and chair-side time. This study aimed to assess and compare the accuracy of LCG calculated using Hanau's formula and right and left lateral records. It also seeks to provide a new formula for calculating LCG.

**Materials and methods:**

An in-vivo, observational, cross-sectional study was conducted with hundred and sixty readings from twenty patients, divided into four groups (forty readings per group): Group A (control)- 2 frontal cephalograms (1st-maximum intercuspation and 2nd-5 mm lateral excursion), Group B- lateral records, Group C- Hanau's formula, and Group D-the new formula. Horizontal condylar guidance (HCG) values from protrusive records were used in Hanau's formula to calculate LCG. LCG values were also obtained from lateral records, frontal cephalograms (taken with lateral jigs), and the new formula. These values were tabulated and statistically evaluated using One-way ANOVA for group comparisons, Tukey's post hoc test for pairwise comparisons, and Shapiro-Wilk test for data normality.

**Results:**

The mean LCG values for Group B were 32.3 (right) and 30.8 (left), and for Group D were 31.15 (right) and 31.75 (left), comparable to Group A of 33.17 (right) and 33.69 (left). The mean LCG values for Groups C were 15.33 (right) and 15.41 (left) and were found to be statistically significant with Group A.

**Conclusion:**

LCG values obtained using the new proposed formula are closer to control values, making it a viable alternative to interocclusal records and more accurate than Hanau's formula.

## 1 Introduction

Condylar guidance is a crucial factor in achieving the desired occlusion scheme in dental prosthetics. Rudolph Hanau emphasized the importance of this guidance in determining the occlusal relationships between the maxillary and mandibular teeth.^1^ According to Hanau, the three types of condylar guidance are lateral guidance (sagittal inclination), anteroposterior guidance (horizontal inclination), and lateral aberration.^1^ Lateral condylar guidance (LCG) is important to be accurately adjusted on the articulator to ensure the reproduction of inclinations that are comparable to the patient's temporomandibular articulation.^2^ Among the articular parameters, canine and molar morphology are strongly influenced by LCG.^3^ Therefore, condylar guidance is essential in determining tooth morphology and avoiding occlusal disturbances. Accurate measurement and transfer of LCG, along with other determinants of mandibular movements, to the articulator are vital in prosthesis fabrication. Patients and the dentist may find it annoying when occlusal interferences during mandibular movements result from inaccurate LCG recording. This increases the amount of chairside time needed for prosthesis correction.

Hanau's formula for lateral condylar guidance, proposed by Sir Rudolph Hanau in 1930, is a mathematical concept used to estimate LCG through its relationship with horizontal condylar guidance. The average LCG is commonly determined using this formula, L = H/8 + 12, where “H" represents the horizontal condylar (protrusive) guidance and “L" represents the calculated lateral condylar guidance.^4^ Hanau suggested this formula as a method for programming articulators.^5^

According to Scandrett FR,^6^ the origin and rationale behind Hanau's LCG equation were never clearly explained by Hanau himself, which raises questions about the equation's validity. Significant changes in horizontal condylar inclination seem to result in only minor alterations in the calculated LCG, suggesting the equation may not be as sensitive as expected.^6^

According to Beu in 1960, Jack Stern who was Hanau's long-term partner disclosed that after 10 years of research (1920–1930), Hanau established that there was a certainly a correlation between the horizontal and lateral inclinations. He observed the lateral settings to constantly range around 15°. Stern claimed that the “Formula” was never deliberated to be exact. Rather, it was an approximate or starting point. Hanau did not want to tell the profession to simply set the lateral controls at 15°. That would suggest a step backwards to an average value instrument. “Hence, Hanau developed the ‘Formula’ as a security blanket”.^7^

Bhawsar et al. compared LCG values produced with Hanau's formula to those acquired with a computerized jaw tracking device (Kinesiograph), finding statistically significant differences between the two methods.^4^ They suggested reevaluating the present average settings and using Hanau's method to program semi-adjustable articulators.

Similarly, Praveena et al. found substantial discrepancies in LCG values computed using Hanau's technique with those obtained from submental-vertex radiography tracings.^8^ This study concluded that radiographic approaches are more reliable and reduce errors in complete dentures caused by calibration and arbitrary formula computations for LCG. Javid and Porter discovered significant discrepancies in condylar readings between lateral interocclusal records and Hanau's formula, supporting the use of lateral interocclusal records for precise restorative procedures.^5^

Given the uncertain dependability of Hanau's formula, lateral interocclusal records appear to be a more reliable method. However, the clinical difficulties associated with obtaining lateral records such as the time-consuming nature of the procedure and the difficulty patients may face in performing the needed movements highlight the need for a more accurate and practical formula.

While obtaining lateral records for every patient is often cumbersome, previous research indicates that Hanau's formula may yield significantly lower lateral guidance values. This study introduces a more accurate alternative, proposing formula L = H/2 + 18 for calculating lateral condylar guidance (LCG) using protrusive records. This approach is designed to enhance accuracy while simplifying the process for both clinicians and patients. Hence, the hypothesis generated for this study was, there is a difference in the lateral condylar guidance obtained by lateral records, Hanau's formula, proposed formula and frontal cephalogram.

The current study's rationale is to analyze and compare lateral condylar guidance calculated by obtaining right and left lateral records, Hanau's formula and the proposed formula utilizing protrusive records, to LCG obtained by frontal cephalogram.

## 2 Materials and methods

A clearance was obtained from the scientific advisory committee and Ethics committee of the Institution (clearance certificate number: SDCH/IEC/2019-20/In/19/33) before commencing the study. A written informed consent was taken from the participants before commencing the study.

### 2.1 Determination of sample size

Sample size was calculated from OpenEpi, Version 3, open-source calculator--SSMean using this formula:

$$\frac{N=\left({\sigma_{1}}^{2}+{\sigma_{2}}^{2}\;/\;\kappa \right)\;{\left(Z_{1–\;\alpha \;/2}+Z_{1\;–\;\beta }\right)}^{2}}{\Delta^{2}}$$

The notations for the formulae are.

N = sample size
σ1 = standard deviation of Group 1
σ2 = standard deviation of Group 2
Δ = difference in group means
κ = ratio = 1
Z_1_ – _α /2_ = two-sided Z value (e.g., Z = 1.96 for 95 % confidence interval)
Z_1_ – _β_ = power.

Eight readings per participant were recorded and one hundred and sixty readings were obtained from twenty participants. Frontal cephalograms, lateral records, Hanau's formula, and the new formula were used to obtain LCG values, which were then compared to the LCG values obtained using Hanau's method. These numbers were totalled and subjected to statistical analysis. Each of the three intervention groups, as well as the single control group, included forty readings per group.

Forty readings each (twenty right and twenty left) of LCG angles were obtained as per following four groups: Group A: LCG obtained using frontal cephalogram (Control group), Group B: LCG obtained using lateral records, Group C: LCG obtained using Hanau's formula, Group D: LCG obtained using the new formula (L = H/2 + 18)

### 2.2 Inclusion and exclusion criteria

The inclusion criteria for participants were; age group between 20 and 30 years, full set of teeth or a single missing tooth and absence of restorations.

Participants with temporomandibular joint disorders, those requiring orthodontic treatment, or those with restorations and fixed or removable partial dentures were excluded.

### 2.3 Procedure for recording the protrusive record

**Fig. 1:**
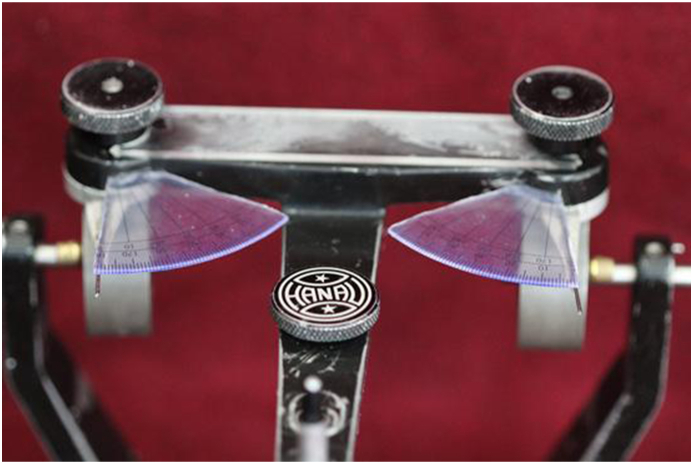
Sectioned protractor attached for modification of articulator.

For accurate evaluation, the LCG readings on the upper member of the Hanau Wide-Vue semi-adjustable articulator (Whip-Mix) were modified. A protractor ranging from 0° to 50° was attached to the Bennett calibrations (Fig. 1), allowing measurement of readings up to 1° precision.

**Fig. 2:**
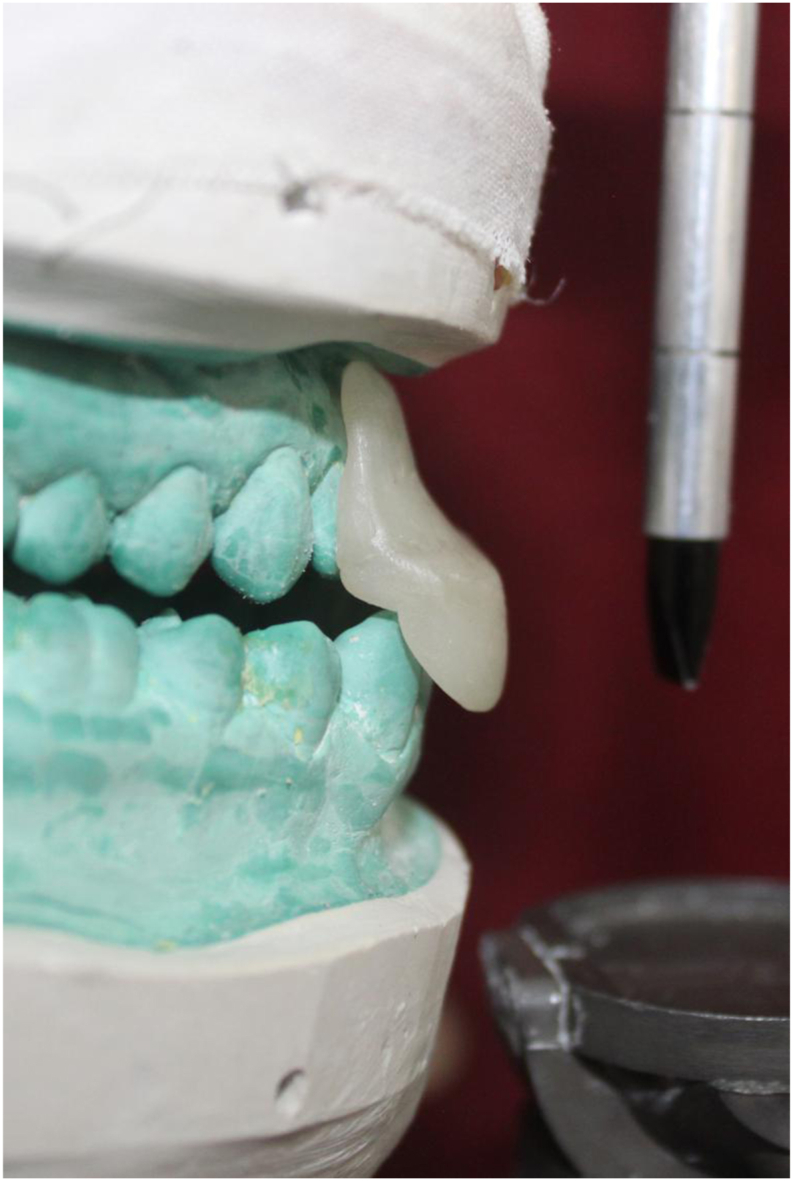
Fabrication of protrusive jig on articulator.

**Fig. 3:**
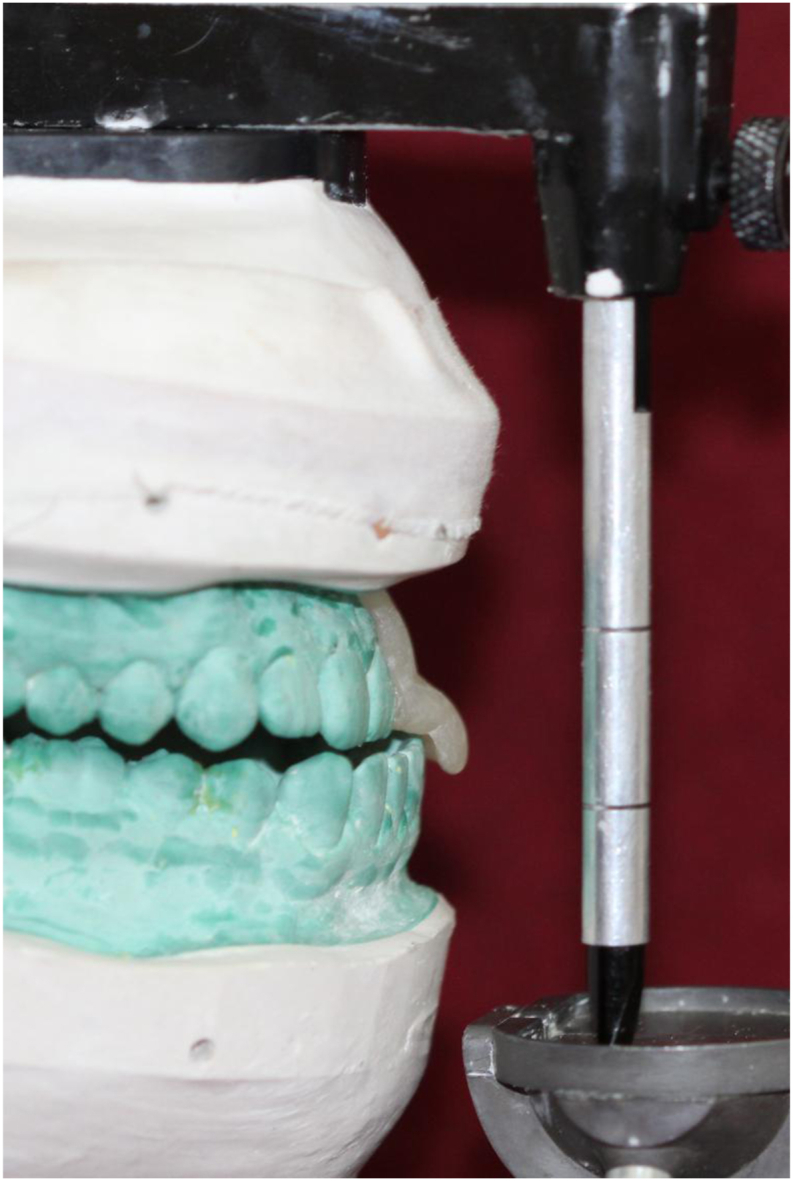
Fabrication of lateral jig on articulator.

**Fig. 4:**
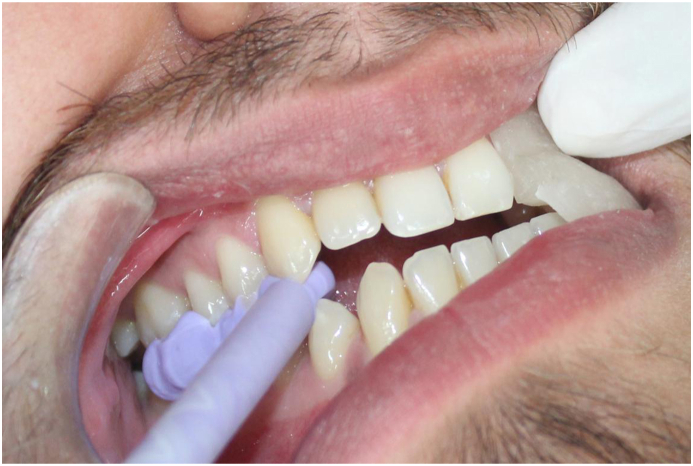
Obtaining lateral records using lateral jig.

On the Hanau Wide-Vue Articulator, a mandibular cast and a maxillary split cast mounted in maximum intercuspation. A customized jig was fabricated by adapting auto-polymerizing clear polymethylmethacrylate (DPI) on the labial surface of maxillary teeth on the cast. This jig assisted in guiding the subjects during mandibular protrusion at 6 mm. Separate jigs for left and right lateral movements were fabricated to guide the subjects laterally at 5 mm. The protrusive jig extended from the right lateral incisor to the left lateral incisor (Fig. 2), while the lateral jigs extended from the canine to the second premolar in the same quadrant (Fig. 3). The horizontal segment of the jig facilitated positioning and retention in the subject's mouth by having indentations of the maxillary teeth. The segment extended up to the mandibular incisal edge, with a 6 mm anterior extension marked and removed. The jigs were designed for creating disocclusion and to guide the mandibular movement in desired manner. Subjects were guided in protrusion until the mandibular incisors touched the anterior projection of the jig, ensuring 6 mm of protrusion. For lateral records, subjects were directed in the right and left lateral movements until the mandibular canines on lateral movement engaged in the grove created at 5 mm distance. (Fig. 4), and records were taken using polyvinyl siloxane bite registration material (Avuebite, Dental Avenue).

### 2.4 Transferring the records to the articulator

The protrusive records were seated between the mandibular and maxillary casts, securing both together. The right and left horizontal condylar guidance (HCG) were modified until the split was properly placed in the notches cut into the maxillary cast. At this stage, the values for the right and left horizontal inclinations were recorded. These HCG values were utilized in Hanau's formula: L = H/8 + 12 to get the right and left LCG. For Lateral records, the centric lock on both the sides and the right Bennett thumbscrew on the articulator were loosened. The obtained left lateral record was seated on the mandibular cast and the maxillary cast was placed according to the indentations obtained. The maxillary and mandibular casts were secured together and made stable. The split was accurately seated in the notches made on the maxillary cast. Once seated properly, the centric locks and LCG thumb screw on right side were tightened. Similar procedure was repeated for recording left lateral condylar guidance using records.

### 2.5 Determining lateral condylar guidance using cephalograms

**Fig. 5:**
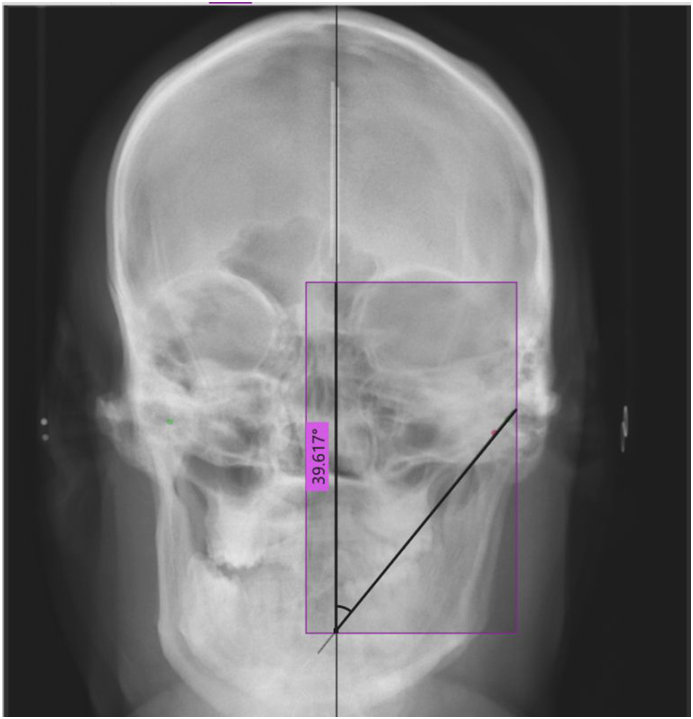
Obtaining Right LCG using cephalogram.

**Fig. 6:**
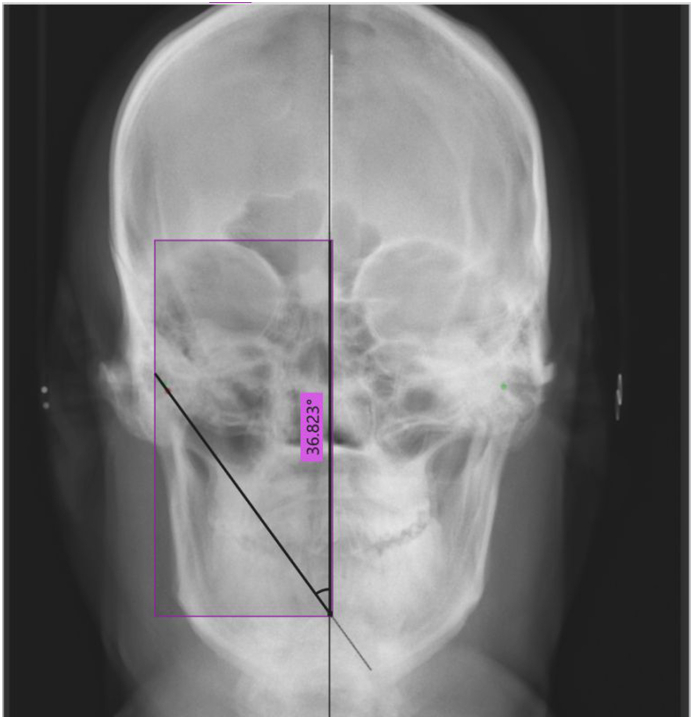
Obtaining Left LCG using cephalogram.

Three digital frontal cephalograms: anteroposterior view (Cephalometric X-ray system. Planmeca) were taken: one in the normal condyle position (maximum intercuspation), one in left lateral, and one in right lateral condylar movement. A central marking scale representing the sagittal axis was placed for ease of superimposition and standardization. The cephalogram were transferred to Adobe Photoshop Version 23.1.1. The highest point on both condyles was marked on the cephalogram with maximum intercuspation. For the right lateral movement cephalogram, the highest point was marked on the left condyle, and vice versa for the left lateral movement cephalogram. The maximum intercuspation cephalogram was adjusted to 40 % opacity and superimposed on the lateral movement cephalograms, ensuring an accurate overlap of the central marking scale. The angle formed by the line passing through the highest points on the condyles and the sagittal axis was marked, representing the LCG. This edited image was transferred to the Microsoft software “Measure Pixels,” the angle was calculated in degrees (Fig. 5, Fig. 6).

### 2.6 Calculating the LCG using proposed formula

LCG values were also calculated using the new proposed formula: L = H/2 + 18. The results from Hanau's formula, lateral recordings, the new proposed formula, and frontal cephalograms were tallied and statistically analysed using One-way ANOVA and Tukey's post hoc test.

### 2.7 Statistical analysis

The study involved 160 readings from 20 participants. LCG values were calculated using lateral and protrusive records with Hanau's formula and compared with those obtained from frontal cephalogram. Data were analysed using IBM Statistical Product and Service Solution (SPSS) Statistics for Windows, Version 28.0, IBM Corp. (2021), Armonk, NY. One-way ANOVA was used for inter-group comparisons, followed by Tukey's post hoc test for pairwise comparisons. Statistical significance was set at *P <* 0.05. Shapiro Wilk test was used for checking normality of data. Data was found to be parametric.

## 3 Results

The results presented in Table 1 revealed significant differences in the left-side LCG values obtained by the four methods—frontal cephalogram, lateral records, Hanau's formula, and the new formula L = H/2 + 18—as demonstrated by the one-way ANOVA F-test (P < 0.001). Pairwise comparisons in Table 2, using Tukey's post hoc test, showed that the frontal cephalogram group (Group A) had higher LCG values than the other groups, although the differences with the lateral records group (Group B) and the new formula group (Group D) were not statistically significant (P > 0.05). However, the frontal cephalogram group did have significantly higher values (P < 0.001) compared to Hanau's formula group (Group C). Lateral records also showed significantly higher LCG values (P < 0.001) than Hanau's formula, while no significant difference was observed between the lateral records group and the new formula group (P > 0.05). Interestingly, the new formula group demonstrated significantly higher values (P < 0.001) compared to Hanau's formula.

**Table 1:** Overall comparative evaluation of lateral condylar guidance - left side obtained by frontal cephalograms, lateral records, Hanau's formula and L = H/2 + 18 respectively.

Left Side	Mean	SD	One-way Anova ‘F’ test	*P* value, Significance
Group A	33.69	4.38	F = 95.484	*P <* 0.001∗∗
Group B	30.8	4.68		
Group C	15.41	1.04		
Group D	31.75	4.14		

∗∗*P <* 0.001 – highly statistically significant difference.

**Table 2:** Pairwise comparative evaluation of lateral condylar guidance -left side obtained by lateral records, Hanau's formula and frontal cephalograms respectively by using Tukey's post hoc test.

Left Side	Comparison Group	Mean Difference	*P* value, Significance
Group A Vs	Group B	2.89	*P* = 0.091(NS)
	Group C	18.28	*P <* 0.001∗∗
	Group D	1.94	*P* = 0.387 (NS)
Group B Vs	Group C	15.39	*P <* 0.001∗∗
	Group D	0.95	*P* = 0.864 (NS)
Group C Vs	Group D	16.34	*P <* 0.001∗∗

*P >* 0.05 – not significant ∗*P <* 0.05 – significant ∗∗*P <* 0.001 – highly significant.

Similarly, the data in Table 3 showed highly significant differences (P < 0.001) among the four study groups for the right-side LCG values. Pairwise comparisons in Table 4 again revealed that the frontal cephalogram group (Group A) had higher LCG values than the other three groups, though differences with the lateral records group (Group B) and the new formula group (Group D) were not statistically significant (P > 0.05). The frontal cephalogram group was significantly higher (P < 0.001) than Hanau's formula group (Group C). Lateral records showed significantly higher values (P < 0.001) compared to Hanau's formula, while the new formula group also had significantly higher LCG values (P < 0.001) than Hanau's formula. No significant differences were seen between the lateral records and the new formula group (P > 0.05). Overall, these findings, as illustrated in Table 1, Table 2, Table 3, Table 4, demonstrate that the new formula produces LCG values closely aligned with those from lateral records and frontal cephalograms, further validating its accuracy and practicality over Hanau's formula.

**Table 3:** Overall comparative evaluation of lateral condylar guidance – right side obtained by frontal cephalograms, lateral records, Hanau's formula and L = H/2 + 18 respectively.

Right Side	Mean	SD	One-way Anova ‘F’ test	*P* value, Significance
Group A	33.17	4.86	F = 68.937	*P <* 0.001∗∗
Group B	32.3	5.22		
Group C	15.33	1.36		
Group D	31.15	5.53		

∗∗*P <* 0.001 – highly statistically significant difference.

**Table 4:** Pairwise comparative evaluation of lateral condylar guidance - right side obtained by lateral records, Hanau's formula and frontal cephalograms respectively by using Tukey's post hoc test.

Right Side	Comparison Group	Mean Difference	*P* value, Significance
Group A vs	Group B	0.87	*P* = 0.931
	Group C	17.84	*P <* 0.001∗∗
	Group D	2.02	*P* = 0.504
Group B vs	Group C	16.97	*P <* 0.001∗∗
	Group D	1.15	*P* = 0.856 (NS)
Group C vs	Group D	15.82	*P <* 0.001∗∗

*P >* 0.05 – not significant ∗*P <* 0.05 – significant difference ∗∗*P <* 0.001 – highly significant.

## 4 Discussion

Dental articulators are essential for accurately reproducing a patient's mandibular movements, crucial for successful occlusion restoration.^4^ Horizontal and lateral condylar guidance, which must be set on semi-adjustable articulators, are typically derived from interocclusal records and Hanau's formula.^4^

LCG and lateral movements are precisely proportional to the lateral side shift. In occlusal rehabilitation, the LCG has great gnathological importance since the value of the angle affects the occlusal relationships of denture fabrication.^9^ The inclined planes of occluding surfaces of teeth affect the movements of the condyles while performing lateral and protrusive movements in dentate subjects.^10^ The direction of the movement of the condyles was altered in case of cuspal attrition or reconstruction of the occluding surfaces.^10^ The more the lateral side shifts, the more distal the working and balancing cuspal slopes of the maxillary posteriors and the lower the cusp height of the mandibular posterior teeth.^9^ Similarly, the cusp height of the posterior teeth increases as the lateral side shift decreases.^9^ All of these facts support the need to evaluate and register LCG.

The newly proposed formula was developed through a critical analysis of the shortcomings of Hanau's original formula, which has traditionally been used to estimate lateral condylar guidance (LCG). However, Hanau's method often yields LCG values that are significantly lower than those obtained from direct and precise clinical measurements, such as frontal cephalograms and lateral records. To address this inconsistency, a series of experimental modifications were made by systematically adjusting the parameters of the original formula.

By altering the ratio between horizontal condylar guidance and lateral condylar guidance, specifically through a reduction in the denominator and a calibrated increase in the additive constant, a revised formula was established. This adjustment resulted in LCG values that demonstrated a much stronger correlation with those obtained from frontal cephalograms and lateral records. The improved congruence suggests that the modified formula provides a more individualized and actual clinical estimation of a patient's LCG.

This closer alignment with imaging-derived and motion-recorded data carries significant clinical implications. It allows for more accurate programming of articulators, leading to better occlusal harmony, functional efficiency, and overall prosthodontic treatment outcomes. By bridging the gap between theoretical estimations and real anatomical and functional data, the revised formula enhances both diagnostic precision and therapeutic predictability.

Frontal cephalograms, widely used in orthodontics and maxillofacial studies, provide a reliable measurement of skeletal structures and their relationships,^11^ making them a suitable reference for refining prosthodontic formulas. The combination of clinical data with these refined calculations improves the precision of LCG values, which is crucial for the customization of prosthetic devices such as dental articulators. Proposed formula, “L = H/2 + 18,” offers a more practical and patient-friendly approach, especially when considering the difficulty of obtaining precise lateral records for every patient. This innovation could lead to more comfortable and efficient treatments, enhancing the overall accuracy of prosthetic rehabilitation procedures while reducing time and complexity for clinicians.

In this study, LCG values were derived using lateral records, Hanau's formula, and a newly proposed formula, and these values were compared with frontal cephalograms to assess the accuracy of these methods. The direct relationship between radiographic condylar guidance tracing and its anatomical equivalent has clinical implications. It was discovered that the data generated from such tracings might be utilized to calibrate the condylar guide settings of semi-adjustable articulators.^8^ As a result, in this investigation, the values obtained from frontal cephalograms were considered optimum.

The lateral condylar guidance values obtained using Hanau's method varied from 13° to 18°, whereas those derived through frontal cephalograms varied from 23° to 45°. Similar studies by Praveena K et al.,^8^ Bhawsar et al.,^4^ and Shetty R et al.^12^ found that LCG obtained using Hanau's formula ranged from 14° to 18°, whereas LCG values obtained using other techniques, such as lateral records and a computerized K7 jaw tracking device, ranged from 7° to 42°. As a result, Hanau's formula yields much lower results for lateral condylar guidance than other regularly used methods for recording lateral condylar guidance in dentulous and edentulous patients.

According to Okeson,^13^ when the mandible moves laterally, the orbiting condyle creates a higher condylar guidance angle than when the mandible protrudes straight ahead. It implies that the lateral condylar guidance is usually greater than the horizontal condylar guidance; which is because the mandibular fossa's medial wall is typically steeper than the fossa's articular eminence anterior to the condyle.^13^

In this study, when Hanau's formula was employed to derive lateral condylar guidance, only two of the 40 readings (20 left and 20 right) exceeded the horizontal condylar guidance values. As a result, the LCG values calculated using Hanau's formula almost always contradicted Okeson's concept.

The results of this study show that the average left and right LCG values derived from Hanau's formula were 15.41 and 15.33, respectively. The new proposed formula resulted in mean left and right lateral condylar guidance values of 31.75 and 31.15, respectively, while frontal cephalograms yielded mean left and right lateral condylar guidance values of 33.69 and 33.17. Twenty-seven of the forty values obtained using the new formula were closer to the control group values, with a difference ranging from 0 to 5°. Twelve of the forty values obtained using the new formula differed by 5.1°–10° from the control group, while one of the forty values differed by more than 10°. The LCG values obtained using the new formula are significantly closer to those obtained using frontal cephalograms.

The new formula for calculating lateral condylar guidance (LCG) offers several significant advantages. It produced values that closely matched those of the control group and demonstrated improved accuracy compared to Hanau's formula, all without requiring additional effort or compliance from patients to record lateral movements. This streamlined approach not only simplifies the clinical workflow but also reduces the potential for errors associated with manual recording. By enhancing the precision of settings on semi-adjustable articulators, the formula contributes to more accurate prosthetic outcomes requiring lesser chair-side occlusal adjustments. Furthermore, the reduced need for patient cooperation makes the process more time saving, efficient and comfortable. As dentistry continues to integrate technology and evidence-based methods, adopting this improved formula could play a key role in elevating both the quality of care and overall patient satisfaction.

The limitation of this study is that the sample is homogenous. According to an article given by Fletcher AM,^14^ a statistically significant difference of 13.6 ± 4.4° in the sagittal condylar guidance angles (SCGAs) between two ethnic groups, indicating notable anatomical differences. These findings have important implications for restorative and prosthetic dentistry, particularly in treatments that require precise use of a dental articulator. To ensure that the newly proposed formula is effective across a broader population, further research is needed. This should involve testing on diverse patient groups, including individuals from various racial and ethnic backgrounds, using semi-adjustable articulators, especially in complex prosthodontic cases. Such inclusive research would improve the reliability and applicability of the formula and contribute to more accurate and personalized prosthodontic care.

## 5 Conclusion

The study concluded that the new formula, L = H/2 + 18, produces LCG (Lateral condylar guidance) values that are significantly closer to those obtained from frontal cephalograms, which is a more precise radiographic method. In contrast, Hanau's formula frequently resulted in lower LCG values, which has led to concerns about its accuracy and reliability when applied in clinical practice. Given these discrepancies, the study suggests that the new formula offers a more dependable and practical approach for determining LCG, thereby reducing the reliance on lateral records. This alternative method also minimizes patient discomfort and chairside time, contributing to a more efficient and patient-friendly dental practice.

## Source(s) of support

Nil.

## Presentation at a meeting

Nil.

## Declaration of generative AI and AI-assisted technologies in the writing process

During the preparation of this work the author(s) used Chat GPT in order to improve the readability and language. After using this tool/service, the author(s) reviewed and edited the content as needed and take(s) full responsibility for the content of the publication.

## Sources of funding

Nil.

## Declaration of competing interest

The authors declare that they have no known competing financial interests or personal relationships that could have appeared to influence the work reported in this paper.
